# PReS-FINAL-2116: Assessment of disease activity by patients with juvenile idiopathic arthritis and the parents compared to the assessment by pediatric rheumatologists

**DOI:** 10.1186/1546-0096-11-S2-P128

**Published:** 2013-12-05

**Authors:** W Armbrust, J Kaak, J Bouma, O Lelieveld, N Wulffraat, P Sauer, E van Sonderen

**Affiliations:** 1 Pediatric Rheumatology, Beatrix Children's Hospital, University of Groningen, University Medical Center Groningen, Groningen, Netherlands; 2 Beatrix Children's Hospital, Groningen, Netherlands; 3 Health Psychology Section, Health Sciences, University of Groningen, University Medical Center Groningen, Netherlands; 4 Department of Rehabilitation, University of Groningen, University Medical Center Groningen, Groningen, Groningen, Netherlands; 5 Pediatric Rheumatology/Immunology, Wilhelmina Children's Hospital, University of Utrecht, University Medical Center Utrecht, Utrecht, Netherlands; 6 Beatrix Children's Hospital, University of Groningen, University Medical Center Groningen, Netherlands; 7 Health Psychology Section, Department of Health Sciences, University of Groningen, University Medical Center Groningen, Groningen, Netherlands

## Introduction

Disease activity in children with juvenile idiopathic arthritis (JIA) is assessed regularly by a rheumatologist. Early detection of disease activity at home, between scheduled consultations, is a major concern. According to current best practice patients with JIA should be treated as soon as symptoms appear. Underestimating disease activity by patients and their parents invariably leads to delayed treatment with joint damage as a consequence. Overestimation may lead to the patient taking less part in sport and leisure activities, missing school, and excessive medication. Therefore it is important to study how patient and parents assess the disease activity compared to the rheumatologists' assessment.

## Objectives

We investigated whether children JIA and their parents are capable of assessing disease activity by comparing their assessments to rheumatologists' assessments. And we studied which factors contribute to the assessment of active disease by the child and parent.

## Methods

Patients and parents assessed 69 joints on a paper homunculus and marked each joint with a different color according to presumed presence of disease: active disease (AD), doubt, and non-active disease (NAD). Their assessments were compared to the rheumatologists' assessments. If patients and/or parents marked on or more inflamed joints, it counted as AD. Pain(measured by an Visual Analogue Scale), functional impairment(measured by CHAQ), age and disease duration were analyzed to differentiate more precise between true and false positive and true and false negative assessments.

## Results

We collected assessments of 113 patients and/or parents. AD was assessed 54 times, 33 of which were true positives. NAD was assessed 23 times, 22 of which were true negatives. Doubt was expressed 36 times, 9 of which were assessed by the rheumatologist as AD. Sensitivity and specificity of AD was 0.77 and 0.31. Disease duration and age did not differ between AD and NAD. Pain and functional impairment scored highest in AD, intermediate in doubt, and lowest in NAD. Pain and functional impairment did not relate to Ad assessed by the rheumatologist

## Conclusion

Patients and/or parents seldom missed arthritis but frequently overestimated disease activity. Pain, functional impairment, disease duration, gender, and age did not differentiate between true and false positives. Patients perceived JIA as active if they experienced pain and functional impairment. To reduce overestimation of the presence of AD we need to improve their understanding of disease activity by teaching them to distinguish between primary symptoms of JIA and symptoms like pain and functional impairment.

## Disclosure of interest

None declared.

